# Identification of Mtb GlmU Uridyltransferase Domain Inhibitors by Ligand-Based and Structure-Based Drug Design Approaches

**DOI:** 10.3390/molecules27092805

**Published:** 2022-04-28

**Authors:** Manvi Singh, Priya Kempanna, Kavitha Bharatham

**Affiliations:** Center for Chemical Biology & Therapeutics, InStem, Bellary Road, Bangalore, Karnataka 560065, India; manvis@instem.res.in (M.S.); kpriyaa97@gmail.com (P.K.)

**Keywords:** Mtb, GlmU, virtual screening, molecular docking, molecular dynamics simulations, ligand growing, pharmacophore

## Abstract

Targeting enzymes that play a role in the biosynthesis of the bacterial cell wall has long been a strategy for antibacterial discovery. In particular, the cell wall of *Mycobacterium tuberculosis* (Mtb) is a complex of three layers, one of which is Peptidoglycan, an essential component providing rigidity and strength. UDP-GlcNAc, a precursor for the synthesis of peptidoglycan, is formed by GlmU, a bi-functional enzyme. Inhibiting GlmU Uridyltransferase activity has been proven to be an effective anti-bacterial, but its similarity with human enzymes has been a deterrent to drug development. To develop Mtb selective hits, the Mtb GlmU substrate binding pocket was compared with structurally similar human enzymes to identify selectivity determining factors. Substrate binding pockets and conformational changes upon substrate binding were analyzed and MD simulations with substrates were performed to quantify crucial interactions to develop critical pharmacophore features. Thereafter, two strategies were applied to propose potent and selective bacterial GlmU Uridyltransferase domain inhibitors: (i) optimization of existing inhibitors, and (ii) identification by virtual screening. The binding modes of hits identified from virtual screening and ligand growing approaches were evaluated further for their ability to retain stable contacts within the pocket during 20 ns MD simulations. Hits that are predicted to be more potent than existing inhibitors and selective against human homologues could be of great interest for rejuvenating drug discovery efforts towards targeting the Mtb cell wall for antibacterial discovery.

## 1. Introduction

Tuberculosis is the major cause of death due to the development of resistance against antibiotics for *Mycobacterium tuberculosis* (Mtb). There is an unprecedented need to develop inhibitors against novel targets that complement existing drugs. Among bacteria, the cell wall of Mycobacterium is unusual as it contains characteristics of both Gram-positive and Gram-negative bacteria [1]. The rigidity and the structural strength of the bacterial cell wall to withstand internal osmotic pressure is imparted by the peptidoglycan layer, one of the three main structural components [2]. Synthesis of the peptidoglycan involves a series of steps, in which the synthesis of Uridine-di-phosphate-N-acetylglucosamine (UDP-GlcNAc) from fructose-6-phosphate is the beginning stage. This UDP-GlcNAc is an essential precursor for the biosynthesis of peptidoglycan and lipopolysaccharide, which are constituents of the bacterial cell wall [3,4].

**Figure 1:**
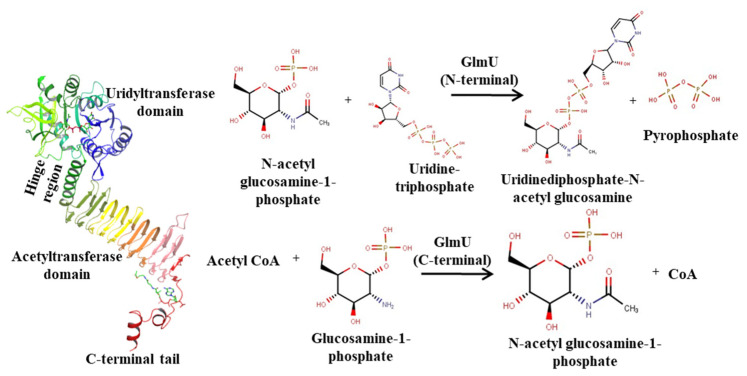
GlmU structure and its reactions. Acetyltransferase reaction catalyzed by C-terminal acetyltransferase domain. Uridyl transferase reaction catalyzed by N-terminal uridyltransferase domain.

N-acetylglucosamine-1-phosphate Uridyltransferase (GlmU) catalyzes two distinct reactions known to act independently [5] (Figure 1). The order of binding in the reaction for Mtb GlmU was proposed based on *Haemophilus influenzae* (HI) as the following: Acetyltransferase activity occurs first at the C-terminal domain where the acetyl group from acetyl-CoA is transferred to the amine group of glucosamine-1-phosphate (GlcN1-P) forming N-acetylglucosamine-1-phosphate (GlcNAc-1-P). GlcNAc-1-P then diffuses to the N-terminal Uridyltransferase active site where, in the presence of UTP, it forms UDP-GlcNAc [6,7]. The UTP and Mg^2+^ initially bind to the Uridyltransferase active site after which the diffused GlcNAc-1-P binds. The enzyme is known to be in open conformation in both apo and UTP bound form. The conformational changes occur when GlcNAc-1-P binds to the N-terminal; the phosphate group of GlcNAc-1-P initiates a nucleophilic attack on the α-phosphate of UTP and catalysis proceeds via a pentacoordinate intermediate at the α-phosphate of UTP stabilized by the Mg^2+^ ion. The final steps include inversion of stereochemistry at the α-phosphate, UTP hydrolysis and release of PPi leading to a product UDP-GlcNAc bound form (closed state). However, a different study has identified that the formation of UDP-GlcNAc is a two-metal-ion dependent process which is involved in the substrate stabilization, nucleophile activation and transition-state stabilization [8]. The order of binding in Mtb in the N-terminal is predicted by the binding of the sugar substrate GlcNAc-1-P and the first ion followed by UTP and the second ion, which differs from the earlier predicted order [6].

GlmU is present exclusively in prokaryotes as a bifunctional enzyme that catalyzes the formation of UDP-GlcNAc [3]. In eukaryotes, Acetyltransferase and Uridyltransferase activity are carried out by GNA1 and UAP1 enzymes, respectively [9,10]. AGX1 and AGX2 are two human UAP1 isoforms resulting from the alternative splicing of a single gene where AGX2 differs in sequence due to the inclusion of an additional 17-amino-acid peptide [11]. These enzymes in eukaryotes share very little sequence similarity with prokaryotic GlmU [9]. Insertional mutagenesis studies using the transposon site hybridization (TraSH) methodology reported that GlmU was an essential gene in Mtb [4]. For Mycobacteria, it was shown that when GlmU was knocked out of *M. smegmatis*, it could not grow without the rescue gene [12]. Another study showed that depleting GlmU Mtb during both normoxic and hypoxic growth resulted in a substantial decrease in cell viability and depletion of GlmU Mtb during ex vivo or in vivo infection either at the start of or after infection has shown clear signs of death of the pathogen [13]. Kinetic modelling of GlmU reactions through computational approaches has shown that inhibition of Uridyltransferase reaction had a maximum impact on the GlmU rate, implying that it is the rate-limiting step [14]. Hence, in this study, we chose to target the Uridyltransferase site for the development of novel inhibitors.

Some inhibitors targeting the Uridyltransferase activities of Mtb GlmU have been reported in recent publications. Few active aminoquinazoline-based compounds with the most potent inhibitor in this series are reported showing an IC_50_ of 74 μM [2]. Later in 2015, structure-based high throughput virtual screening was used that gave diverse compounds, out of which compound 4 showed promise with an IC_50_ of 42.07 μM in ITC [13]. The binding energy of compound 4 to the uridyltransferase active site of Mtb GlmU was similar to that of substrate UTP (K_d_ = 5.8 × 10^−1^ μM of UTP). Soni et al. also reported a novel allosteric inhibitor, Oxa33, targeting the uridyltransferase activity of Mtb GlmU with an IC_50_ of 9.96 µM and inhibited the in vitro growth of Mtb H37Rv with an MIC of ~75 μM [13].

In the present work, we aim to design novel small molecule inhibitors against the Uridyltransferase active site of Mtb GlmU through computer aided drug design approaches. The following strategies were employed: (a) Structural analysis of Mtb and human enzymes for pocket similarity and differences to develop selective inhibitors, (b) optimization of previously known bacterial GlmU inhibitors for Mtb GlmU using the ligand growing approach and bioisosteric search by accounting for the conformational changes upon substrate binding, (c) identification of hits by ligand-based virtual screening through molecular docking and E-pharmacophore methods, and (d) triaging of hits by molecular dynamics simulations of top poses. Hits predicted to be potent and selective could rejuvenate antibacterial discovery efforts by targeting the Mtb GlmU protein.

## 2. Results and Discussions

### 2.1. Pocket Comparison between Mtb GlmU Uridyltransferase and Human Homologs

**Figure 2:**
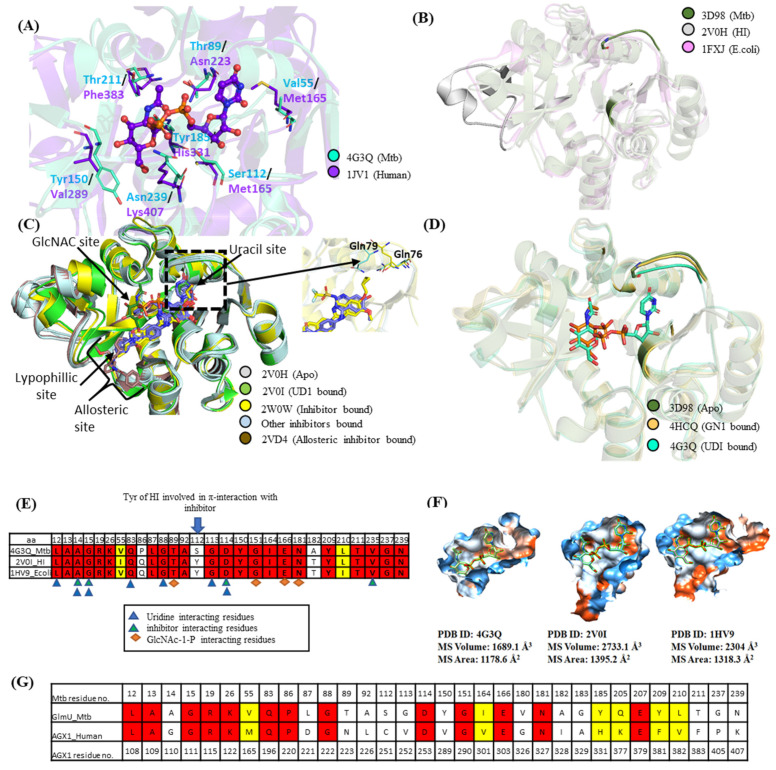
(**A**) Comparison of MtbGlmU with Human AGX1. (**B**) Structural alignment of apo forms of Mtb (PDB ID: 3D98), HI (PDB ID: 2V0H) and *E. coli* (PDB ID: 1FXJ). (**C**) Structural comparison of apo, UD1 and inhibitor bound HI structures. (**D**) Alignment of Mtb GlmU structures; the three forms apo (PDB ID: 3D98), closed (PDB ID: 4G3Q) and GlcNAc-1-P bound (PDB ID: 4HCQ) structures are aligned (**E**) Structure based sequence alignment of pocket residues of N-terminal GlmUMtb with HI and *E. coli*. (**F**) The pocket volume of closed forms of Mtb, HI and *E. coli* generated using CASTp server [17] visualised by MSMS package of Chimera [18]. (**G**) Pocket residues alignment between Mtb and human AGX1. Differing pocket residues of Mtb and human AGX1 could be used in exploring selectivity.

Despite growing evidence of the biological significance of targeting Mtb GlmU Uridyltransferase activity, no potent inhibitors have been developed so far due to the fear of hitting homologous human enzymes. To identify homologous human enzymes, its structure was screened against the DALI database and Site2Vec algorithm [15,16]. Two human proteins, AGX1 and AGX2, UDP-N-acetylglucosamine (UDPGlcNAc) pyrophosphorylase (AGX1 and AGX2 differ by an alternately spliced 17 residues of peptide and both AGX1 and AGX2 bind to their substrate similarly) have high structural homology to the Mtb GlmU Uridyltransferase domain with low (17% with AGX1) sequence similarity. Alignment of pocket residues between Mtb and human AGX1 is shown in Figure 2A. A thorough pocket comparison between Mtb GlmU and Human AGX1/AGX2 showed that the nucleotide and sugar binding mode in Mtb GlmU is comparable to the human AGX1 with few dissimilar residues—namely, V55/M165, T89/N223, S112/C251, Y150/V289, Y185/H331, T211/F383 and N239/K407, respectively (Figure 2G). Inhibitors that engage these distinct residues will drive selectivity against human AGX1/AGX2 proteins, thereby encouraging us to identify potent inhibitors for the Mtb GlmU Uridyltransferase domain. 

### 2.2. Pocket Comparison among Bacterial GlmU Uridyltransferase Domain

To date, reported Mtb GlmU Uridyltransferase activity inhibitors have single to two-digit micromolar activity [2,13], while potent inhibitors are available for other bacterial GlmU Uridyltransferase domains. Existing inhibitors for HI [19,20], *E. coli* [19,20], *Xanthomonas oryzae* [21,22] and Mtb [2,13] were collated. Among them, the crystal structures of the GlmU Uridyltransferase domain were solved for HI, *E. coli* and Mtb with various loop differences in apo forms as can be seen in Figure 2B. Structural differences and pocket comparison among them were examined to strategize and predict if the reported inhibitors could inhibit Mtb GlmU. 

HI GlmU is well studied compared to its bacterial orthologues. Alignment of the HI GlmU crystal structures of apo, UMP, UDP and UD1 bound forms (PDB ID: 2V0H, 2V0J, 2V0K, 2V0I) [7] show that major conformational change spanning residues L133–G140 and V150–K166 is observed only in the product UD1 bound form, which induces a closed conformation of the enzyme, as shown in Figure 2C. There are eight inhibitor bound crystal structures reported for HI GlmU (PDB ID: 4E1K [19], 4KNR, 4KNX, 4KPX, 4KPZ, 4KQL [20], 2W0V, 2W0W) where all the inhibitors occupy part of the UTP site, skirts the outer perimeter of the GlcNAc-1-P (N-acetylglucosamine-1-phosphate) pocket and anchors a hydrophobic moiety into a lipophilic pocket. Overall, the pocket could be subdivided into GlcNAc site, Uridine site and lipohilic site as illustrated in Figure 2C. The comparison of the inhibitor bound structures with open apo form (PDB ID: 2V0H) and product bound closed form (PDB ID: 2V0I) shows that the inhibitor bound forms resembles the open form. It is believed that upon inhibitor binding, the conformer is locked in apo form and does not allow UTP binding [19]. Among the inhibitor bound structures, minor ligand-induced conformational changes can be observed. The ligand in PDB ID: 2W0W induces conformational changes similar to the product UD1 bound form (Figure 2C). In some, the orientation of residue Gln-79 appears to be dependent on the type of compound bound. In the inhibitors that occupy the uridine site extending from C7 (replacing hydroxy) or C6 of the ribose ring, the orientation of the Gln-79 residue faces outward, possibly to avoid clashes (PDB ID: 2W0V, 2W0W, 4KNX, 4KPZ, 4KQL). On the other hand, in ligands that do not occupy the uridine site, such as PDB ID: 4E1K, 4KNR, the orientation of residue Gln-79 faces inward (Figure 2C). The changes can also be observed for the Gln-76 residue.

Comparison of the three forms of Mtb GlmU structures, the apo form (PDB ID: 3D98, 3DK5, 3FOQ) [6,23,24], product bound form (UDP-GlcNAc bound, PDB ID: 3D8V, 3DJ4, 3SPT, 3ST8, 4G3Q, 4G3S, 4G87) [6,8,23] and the sugar substrate, GlcNAc-1-P bound form (PDB ID: 2QKX, 4HCQ) [6,8] are similar without major conformational changes, as seen in HI except for the minor loop movement near the UTP site in order to make interactions with uracil moiety through Gln83 and Gly88 (Figure 2D).

Sequence similarity between the Mtb GlmU Uridyltransferase domain with HI an d*E. coli* is 54.1% and 55.7%, respectively. Structure-based sequence alignment highlights the pocket residue conservation, and 4 Å around UDP-GlcNAc is shown in Figure 2E. Comparison of the closed form pocket volume using the CASTp server [17] and MSMS package of Chimera [18] shows that the pocket varies in size as shown in Figure 2F. The pocket volume of HI and *E. coli* is larger than Mtb, suggesting that the GlmU Uridyltransferase inhibitors of HI and *E. coli* might not make the same contacts in Mtb. The structural features defining differences in pocket volume are present in the regions of residues 16–19, 82–87 and 143–184, as highlighted in Figure 2B. To propose potent and selective Mtb GlmU inhibitors, two strategies were applied: (i) optimization of existing bacterial GlmU Uridyltransferase domain inhibitors, and (ii) ligand/structure-based virtual screening.

### 2.3. Optimization of Known Bacterial GlmU Inhibitors for Mtb GlmU

**Figure 3:**
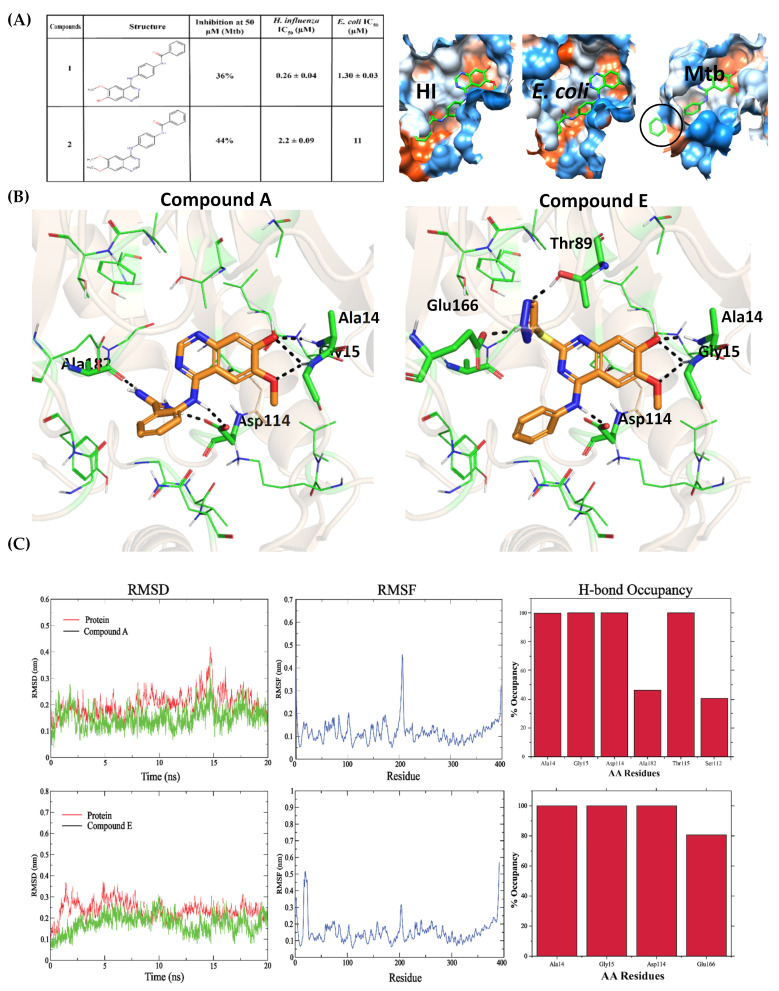
(**A**) The two compounds which were tested on all three organisms Mtb, HI and *E. coli* [2,19]. Compound 1 superposed on to *E. coli* and MtbGlmU pocket. The pocket volumes of apo forms of three organisms generated using CASTp server [17] visualised by MSMS package of Chimera [18]. (**B**) Binding poses of compound A and compound E. (**C**) MD simulation analysis of compound A and E.

**Figure 4:**
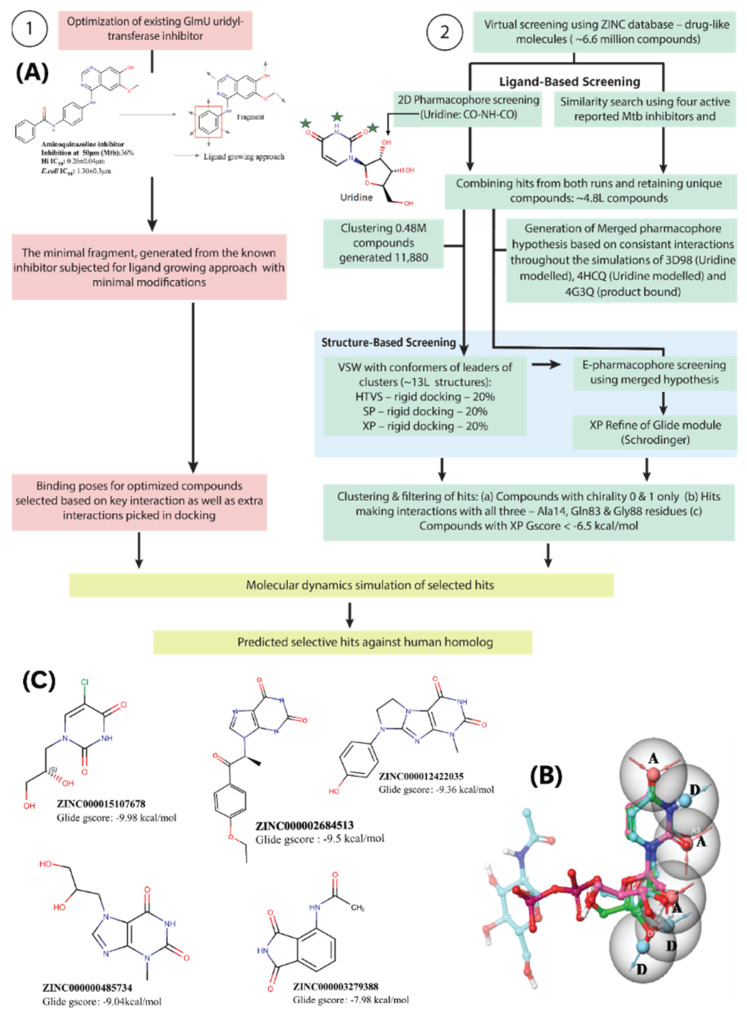
(**A**) Optimization and Virtual screening workflow followed in the present study. (**B**) Generation of merged pharmacophore. The merged hypothesis from all three PDBs:ADAADD. (**C**) Five hits obtained from virtual screening.

A number of Aminoquinazolines and benzamide inhibitors were reported for the Uridyltransferase activities of HI GlmU [19,20]. Only two Aminoquinazoline inhibitors (Figure 3A) designed for HI and *E. coli* were tested for Mtb and showed reduced activity [2]. Figure 3A also shows compound 1 (inhibitor of HI) superposed onto the *E. coli* and Mtb GlmU pocket, and the pocket volume clearly shows that the pocket is significantly smaller in Mtb than HI and *E. coli*. Hence, we explored the minimum compound fragment that could bind to MtbGlmU (Figure 4A). This fragment docked well into 3D98 (Mtb apo form) and retained interactions with Ala14, Gly15 and Asp114, similar as in HI, and contributed −6.1 kcal/mol glide gscore. The fragment was subjected to the ligand growing approach using SPARK/Cresset at the ortho, meta and para positions of the phenyl ring and hydrogen of aminoquniazoline ring with minimal modifications (Figure 4A). The resulting structures were later sampled for various conformations and aligned using Forge. Molecular docking of these designed compounds in the open form of MtbGlmU (PDB ID: 3D98) was performed and the top compounds from each group, which showed a higher glide gscore (Compounds A to H), were analyzed (Figure S1).

The aminoquinazoline with -NH in all the compounds makes H-bond interactions with Ala14, Gly15 and Asp114. Compounds A and B are fragments decorated at ortho positions with (aminomethylidene) azanium and amino(methoxy)methanol, respectively. Compounds A and B make additional H-bond interactions with Gly149 and Ala182 and Gly149 and Glu166, respectively. Compound C has 2-methylpropan-1-aminium attached at the meta position, making H-bond interaction with Gly149 and Glu166. The hydroxy group is attached at the para position to make Compound D, where it formsan H-bond with Asn239. Compound E is grown at the C3 of aminoquinazoline by attaching 1,2,3-triazolidine-4-thiol, which makes H-bond interactions, with Thr89 and Glu166. Compound F has (oxolan-2-yl) methanol replacing hydroxy group and retains the interaction with Ala14, Gly15 and Asp114. Ethane-1,2-diol replaces methoxy in Compound G in which both oxygens make H-bonds with Gly15. In Compound H, the phenyl ring was replaced by 1,2-dihydropyridin-2-ol where an H-bond is formed with Gly149 and Asn239. The binding poses for these compounds are shown in Figure 3B and Figure S2. These top eight compounds were further subjected to MD simulations. The overall optimization workflow is shown in Figure 4A as strategy 1.

### 2.4. Ligand-Based Virtual Screening

Virtual screening of the ZINC database was performed in parallel by ligand-based screening followed by structure-based screening (Figure 4A, strategy 2). Since the compound library is huge, ligand-based screening by 2D pharmacophore and similarity search helped to eliminate and narrow down the inhibitor search space. The features of the uracil group of UTP, which made interactions with Ala14, Gly88 and Gln88 of Mtb GlmU, could be a potential substructure of any GlmU inhibitor. The feature query (SMILES pattern O=CNC=O) was screened with a cut-off of 0.5 Tanimoto coefficient against the ZINC database with a bit-MACCS key molecular fingerprint. Independently, a similarity search was performed for known Mtb inhibitors to find structurally similar compounds that have similar properties. In order to choose a diverse set of reference ligands, we clustered 28 known Mtb GlmU inhibitors and selected the one with the highest inhibition percentage (>40%) from each cluster (Table S1). A structurally diverse set of reported inhibitors was used as query compounds (Table S1; highlighted in bold) to retrieve all possible structures similar to the active inhibitors. Overall, we selected five compounds, including the substrate, UTP, for a similarity search against ~6.6 million ZINC compounds. The similarity was measured by a bit-MACCS molecular fingerprint, and the search was performed with a cut-off of 0.6 Tanimoto coefficient against the entire database. The hits from ligand-based approaches were merged and duplicates were eliminated, resulting in ~480,000 compounds. The clustering of ~480,000 compounds generated 11,880 clusters and the leaders of these clusters were further subjected to structure-based screening.

### 2.5. Structure-Based Virtual Screening through Molecular Docking and E-pharmacophore Methods

Considering differences in the Mtb GlmU Uridyltransferase active site, we chose all three forms for virtual screening. The three Mtb GlmU structures (PDB ID: 3D98, 4HCQ, 4G3Q) were prepared and Uridine/UDP-GlcNAc were docked in the active site of three structures to validate the docking protocol. Among all the UDP-GlcNAc bound co-crystal structures, residues Gly-88, Gln-83, Ala-14, Gly-15 and Asp-114 were involved in H-bond interactions with the uridine part of the UDP-GlcNAc. The top uridine dock poses retained interactions with all the key residues similar to product bound structures with −5.6 kcal/mol and −6.1 kcal/mol glide score in apo and GlcNAc-1-P bound Mtb structures, respectively. UDP-GlcNAc docking resulted in a −14.92 kcal/mol glide score with an RMSD value of 0.58 Å with respect to its co-crystal conformation, suggesting that the protocol can provide reliable hits.

The structure-based virtual screening workflow was performed using two approaches, Molecular Docking and E-pharmacophore, as shown in Figure 4A strategy 2. For molecular docking, the clustered 11,880 leaders’ structures obtained from ligand-based screening of Zinc database were taken forward in three stages, High-Throughput Virtual Screening, Glide SP and Glide XP docking. The hits obtained were filtered based on chirality 0 to 1, the existence of three key interactions with Ala14, Gln83 and Gly88 and a glide XP score < −6.5 kcal/mol that lead to 191 hits. 

In parallel, structure-based screening by E-pharmacophore was strategized. MD simulations of (i) uridine docked in apo form (PDB ID: 3D98), (ii) uridine docked in GlcNAc-1-P bound form (PDB ID: 4HCQ) and (iii) a UD1 bound co-crystal structure (PDB ID: 4G3Q) were performed to quantify the crucial interactions to develop critical pharmacophore features (MD data shown in Figure S3). The C2-/C4-carbonyls and N3 of Uracil make consistent hydrogen bond interactions with Gln83, Gly88 and Ala14 in the three complex structures, while the uridine ribose hydroxyl’s interactions vary between the uridine and the product, UD1 bound forms. The ribose C4 acts as a hydrogen bond acceptor or donor with Gly15 and Leu12, while the ribose C3 hydroxyl acts as a hydrogen bond acceptor or donor with Asp114 and Lys26. Therefore, three features which represent the interactions made by the ribose hydroxyl C3 and C4 were considered. Overall, the merged hypothesis with ADAADD features was chosen representing all the interactions made by uridine (Figure 4C) and screened against the Zinc database. The hits obtained were filtered using the same filters mentioned for structure-based virtual screening, which resulted in 636 hits. 

Based on key residual interactions and visual inspection, a total of 26 hits were selected. Furthermore, these compounds were evaluated for their stability in maintaining the contacts within the pocket by performing MD simulations.

### 2.6. Triaging Hits by MD Simulations of Top Poses

The 20 ns MD simulations performed for uridine docked in apo and GlcNAc-1-P bound crystal structures and UD1 co-crystal structure (PDB ID: 3D98, 4HCQ, 4G3Q) were stable by maintaining all the key uridine interactions (Figure S3). Similarly, simulations were performed for dock poses of ligands obtained from three approaches: (i) ligand growing, (ii) molecular docking and (iii) E-pharmacophore screening.

The binding poses of compound A and compound E (Figure 3B) that developed from fragment optimization were stable throughout the simulation with RMSD of 2.5 Å and 3 Å, respectively, (Figure 3C) when compared to the dock pose. Compound A retained H-bond interactions with Ala14, Gly15 and Asp114 with ~100% occupancy. H-bond occupancy with Ala182 was only 46%, but the amino group had gained H-bond interaction with Thr115 with ~100% occupancy. It also showed 40.61% H-bond occupancy with Ser112 (Figure 3C). In the case of compound E, it showed 100% H-bond occupancy with Ala14, Gly15 and Asp114, but showed 80.57% occupancy with Glu166. Thr89 interaction was lost during the simulation (Figure 3C).

**Figure 5:**
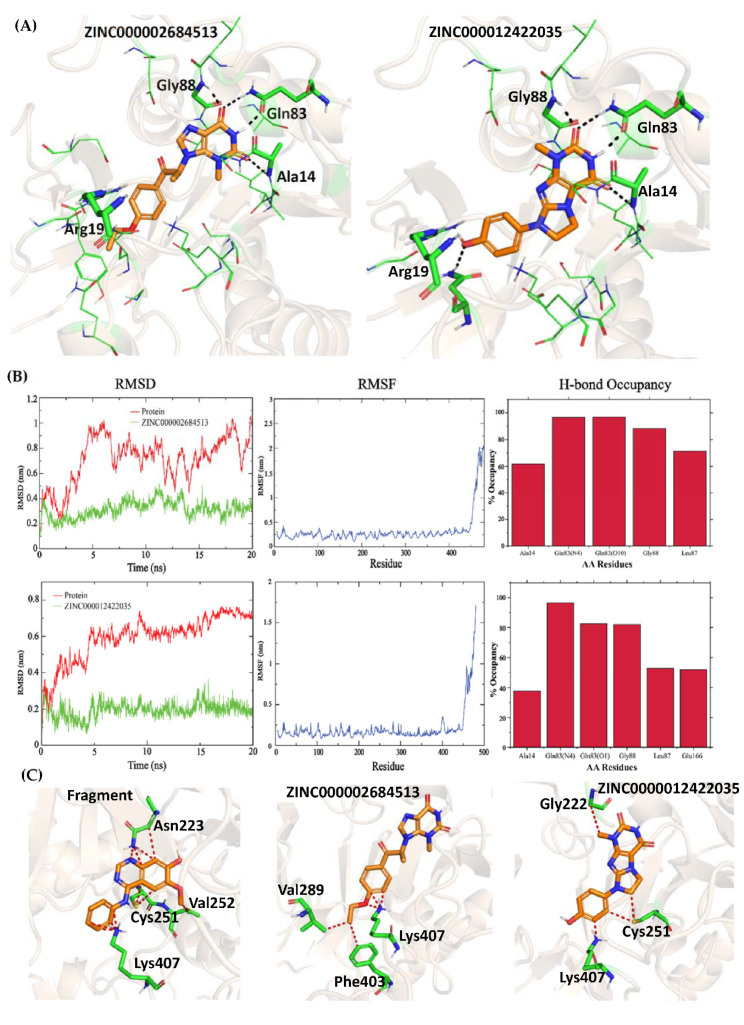
(**A**) Putative binding poses of vs. hits ZINC000002684513 and ZINC000012422035 (**B**) MD simulation of ZINC000002684513 and ZINC000012422035 for 20ns. (**C**) Overlay of Fragment and the three vs. hits ZINC000002684513, and ZINC000012422035 in human AGX1 (PDB ID: 1JV1). Ugly clashes are shown in red color.

MD simulation of 26 hits obtained from the structure-based screening resulted in five hits, which showed stable key interactions during simulations. These five hits are ZINC000015107678, ZINC000002684513, ZINC000012422035, ZINC000000485734 and ZINC000003279388. All these compounds showed key interactions with Ala14, Gln83 and Gly88 in the docking studies (Figure 5A and Figure S4a). The hydroxyls of the ZINC000015107678 compound showed H-bond interactions with Leu12, Gly15 and Asp114 and -Cl showed halogen bonding with Glu207 in docking, whereas during simulation Ala14 and Gly88 showed H-bond occupancy of 100% and 49.3%, respectively. Gln83 showed H-bond occupancy of 80.5% and 58.8% with pyridinone-NH and -CO, respectively. Gly15 and Asp114 were observed with 88.6% and 89.3% occupancy, and halogen bonding with Glu207 was also affected throughout the simulations (Figure S4b). While in docking, the phenyl ring in ZINC000002684513 showed that the π-stack with Arg19 during the simulation of the three key interactions with Ala14, Gln83 and Gly88 were retained with 61.8%, 97% and 88.5% occupancy, respectively, and the additionally gained Leu87 interaction was retained with 71.3% occupancy, but the Arg19 interaction was inconsistent (Figure 5B). In the docking study, the phenol ring of the ZINC000012422035 compound showed H-bond interactions with Asn239 and π-stack with Arg19, whereas in simulation these two interactions were lost but gained Glu166 interaction with 52% H-bond occupancy. The three key interactions with Ala14, Gln83 (pyridinone-NH), Gln83 (pyridinone -CO) and Gly88 were observed with 37.7%, 96.6%, 82.5% and 82% H-bond occupancy (Figure 5C). In docking the hydroxyls of the ZINC000000485734 compound showed H-bond interactions with Ser112 and Asp114, but during simulation the interactions with Gln83 (pyridinone -NH), Gln83 (pyridinone -CO), Gly88 and Asp114 were retained with 91.1%, 99.3%, 90% and 100% occupancy, respectively, whereas the Ala14 interaction was inconsistent with 56.7% occupancy. Leu87 and Ser112 interactions were also observed with 48.5% and 63.3% H-bond occupancies, respectively (Figure S4b). While amide -CO of compound ZINC000003279388 picked H-bond interaction with Lys26, this interaction was lost during simulation, but the compound picked consistent Gly15 interaction with 100% occupancy and also showed 42.6% interaction with Leu87. All three key interactions with Ala14, Gln83 (pyridinone-NH), Gln83 (pyridinone -CO) and Gly88 showed 100%, 99.8%, 79.9% and 95.8%, respectively (Figure S4b). Therefore, compounds ZINC000015107678, ZINC000002684513, ZINC000012422035, ZINC000000485734, and ZINC000003279388 can be used as novel compounds targeting the GlmU uridyltransferase site.

### 2.7. Selectivity against Human AGX1

The structural and sequence differences in the pocket residues of Mtb GlmU and human homolog AGX1 were explored to see if these differences could contribute to the selectivity of the obtained Mtb GlmU hits. Although the nucleotide and sugar-binding mode in both Mtb GlmU and human AGX1/AGX2 are comparable, a few unconserved residues—namely, V55/M165, T89/N223, S112/251, Y150/V289, Y185/H331, T211/F383, and N239/K407—between Mtb and human, respectively, will drive selectivity. Apart from these differing pocket residues, there are a few conserved substituted pocket residues such as G113/Val252, G183/A329 and L210/V382 in Mtb and human, respectively, that would also be useful in exploring selectivity.

To probe if all the hits obtained from fragment optimization and virtual screening were selective against human homolog AGX1, validated binding modes of hits were overlayed onto the human AGX1 substrate binding pocket. The fragment selected for the ligand optimization when positioned onto the human AGX1 pocket itself showed steric clashes with pocket residues such as Asn223 (Thr89 in Mtb), Cys251 (Ser112 in Mtb), Val252 (Gly113 in Mtb), and Lys407 (Asn239 in Mtb) (Figure 5C). Moreover, the substitutions at the Ortho position, aminomethylidene azanium in compound A and 1,2,3-triazolidine-4-thiol in compound E showed clashes with Ala329 (Gly183 in Mtb GlmU) and Ala329/Val382 (Gly183/Leu210 in Mtb GlmU) in human AGX1, respectively. Similarly, the hits obtained from virtual screening, when overlaid onto the human AGX1 pocket, showed clashes with the pocket residues. The ZINC000002684513 compound showed clashes with Val289, Phe403 and Lys407 and ZINC000012422035 with Cys251 and Lys407 (Figure 5C). On the other hand, ZINC000015107678 and ZINC000000485734 showed major clashes with Cys251, and ZINC000003279388 showed clashes with Cys251 and Val252 (Figure S5). Overall, compounds designed based on fragment and virtual screening can be used as starting points to develop selective and potent inhibitors of Mtb GlmU Uridyltransferase.

## 3. Materials and Methods

### 3.1. Protein Preparation

Mtb GlmU structures, apo conformation (PDB ID: 3D98) [6], substrate bound (PDB ID: 4HCQ) [8] and product bound conformation (PDB ID: 4G3Q) were downloaded from the RCSB PDB site [25] and prepared using the protein preparation wizard of Schrödinger [26,27]. The structures were preprocessed via assigning bond orders, adding hydrogens, creating zero bond orders to metals and filling missing side chains using Prime. The water molecules were not deleted during this process. Protonation states for the ligands were generated at pH 7.0 ± 3.0. The protein’s hydrogen bond network was optimized using the H-bond assignment option under the refine tab. Finally, restrained minimization was performed to these structures, which allowed hydrogen atoms to be freely minimized, while allowing for sufficient heavy-atom movement to relax strained bonds, angles, and clashes. The minimized structures were used for docking analysis.

### 3.2. Receptor Grid Generation

Since there is no UTP bound structure available for Mtb GlmU, all the UDP-GlcNAc bound structures of GlmU were aligned and analyzed to study the crucial interacting residues. After analyzing the structures, the Gly-88, Gln-83, Ala-14, and Gly-15 residues of GlmU were observed to be consistently interacting with UDP-GlcNAc. Based on this observation, these residues were used to generate the grid for apo and GlcNAc-1-P bound GlmU protein using the Glide receptor grid generation module. Waters were deleted before grid generation. The potential of nonpolar atoms of the receptor was softened using the Van der Waals radius scaling with scaling factor 1.0 and partial charge cutoff 0.25. No constraints were defined for the apo and GlcNAc-1-P bound GlmU structures during grid generation. In the case of the UDP-GlcNAc bound structure of GlmU (PDB ID: 4G3Q), the grid was defined using the co-crystallized ligand. Two water molecules (HOH 1137 and HOH 1138) interacting with metal were retained in the active site during grid generation. Metal constraint was defined in the grid generation.

### 3.3. Molecular Docking

The Glide module of Schrödinger (Glide, Schrödinger, LLC, New York, NY, USA, 2018) [28,29] was used for docking. The uridine and UDP-GlcNAc structures were drawn using the Marvin sketch (Marvin 19.16.0, 2019, ChemAxon (http://www.chemaxon.com accessed date: 16 September 2019)). The ligand preparation and conformational sampling of uridine was performedas mentioned in the Ligprep and confgen sections, respectively. Uridine conformers were docked in apo and GlcNAc-1-P bound GlmU structures using Glide SP rigid protocol. UDP-GlcNAc conformers were docked in the UDP-GlcNAc bound GlmU structure. The potential of nonpolar atoms of the ligand was softened using the Van der Waals radius scaling with scaling factor 0.80 and partial charge cutoff 0.15. Metal constraint was used during docking. Finally, post docking minimization was performed to optimize the ligand geometries.

### 3.4. Ligand Growing

Spark 10.5.6 software (Cresset^®^, Litlington, Cambridgeshire, UK) [30] was used for the ligand growing approach. The fragment of the known aminoquinazoline Mtb GlmU inhibitor (predicted in this study) was loaded into the software with a 20% score weight and apo GlmU protein 3D98 was used as the excluded volume. The hydrogens at ortho, meta and para positions of the phenyl ring and hydrogen of the aminoquinoline ring of the fragment were selected for the ligand growing approach. The phenyl, hydroxy and methoxy groups were also selected for the ligand growing approach. Spark search was performed against the databases: ChEMBL_common, ChEMBL_rare, ChEMBL_veryrare, Commercial fragments (VeryCommon, Common, LessCommon, Rare), Cresset reagents (eMolecules) and VEHICLe, which gives a 30 databases overall with 885,777 fragments. Fragments with correct angles and distances which matched geometrically were merged in the selected regions and scored against fragment query for structure and shape similarity by Spark software.

The generated structures from Spark were subjected to Forge 10.6.0 (Cresset^®^, Litlington, Cambridgeshire, UK) for the conformational search and their alignment after deleting unstable compounds. Forge software aligns and scores molecules using their electrostatic and shape properties. The results from Forge were further docked into 3D98 apo form (Zhang et al., 2009) [6] using the SP rigid protocol of the Glide module of Schrödinger (Glide, Schrödinger, LLC, New York, NY, USA, 2018, [28,29]).

### 3.5. ZINC Database

The SMILES of drug-like molecules (9,472,331) were downloaded from the ZINC15 database [31]. After removing duplicates, 6,620,478 unique SMILES were obtained (596 SMILES out of ~9.4 million were not processed as there was no structure information available). The physicochemical properties of ~6.6 million compounds were generated using Canvas to understand the range of these properties [32,33,34].

### 3.6. Ligand Preparation

The Ligprep module of Schrödinger (LigPrep, Schrödinger, LLC, New York, NY, USA, 2018) was used to convert 2D ligand structures to 3D structures. The ligands were processed to assign the suitable protonation states at physiological pH 7.4 ± 0.0 using Epik. Specified chiralities were retained. The generated ZINC database compounds, uridine, UDP-GlcNAc and reported Mtb GlmU inhibitors were subjected to Ligprep. Uridine chirality was retained as present in the UDP-GlcNAc structure.

### 3.7. Conformational Sampling

The Confgen module of Schrödinger (ConfGen, Schrödinger, LLC, New York, NY, USA, 2018, [35]) was used for conformer generation using OPLS 2005 force field. The default setting was used for conformational sampling. The ligprep outputs of the ZINC database compounds, uridine, UDP-GlcNAc and reported Mtb GlmU inhibitors were subjected to Confgen.

### 3.8. Ligand-Based Screening

Ligand-based screening was conductedin Molecular Operating Environment (MOE) 2018.01 [36] in two ways:

(1) 2D-pharmacophore based screening using SMARTS pattern O=CNC=O of the uridine structure (which makes key interactions with residues Ala14, Gly88 and Gln88) against ~6.6 million ZINC compounds with 0.5 tanimoto coefficient (TC). Initially before the screen, known Mtb inhibitors were randomly seeded with the ZINC database and used for the 2D pharmacophore similarity search using MOE with both 0.5 and 0.4 TC. However, with 0.4, we obtained 27 Mtb inhibitors out of 37 (including active and inactive compounds) and with 0.5, none of the Mtb inhibitors were obtained in the screened hits. We chose 0.5 TC since all the Mtb inhibitors were obtained when we performed a similarity search with four known active Mtb inhibitors.
(2) A similarity search was performed with a diverse set of known Mtb inhibitors and the substrate UTP against ~6.6 million ZINC compounds with 0.6 TC. In order to choose a diverse set of reference ligands, we clustered the known inhibitors and selected the one with the highest inhibition percentage from each cluster (201305, 201504, 201506, 201507; name is assigned based on the year of publication and the compound no. given in the respective article—for example, 201305: 2013 is year and 05 is compound no.). Additionally, we included UTP since we are targeting the UTP site. Hence, we selected five compounds for the similarity search.

Both screenings were conducted using the Bit MACCS fingerprint of MOE. Hits obtained from both screenings were merged and unique hits of ~4.8 lakhs compounds were retained.

### 3.9. Clustering

Binary fingerprints for ~4.8 lacs compounds obtained from ligand-based screening were generated using atom-pairs fingerprints of Canvas/Schrödinger [32,33,34]. The leader-follower method of Canvas/Schrödinger was used for clustering, which led to 11,880 leaders. These compounds were submitted for Ligprep and further conformational sampling was performed using Confgen.

### 3.10. Virtual Screening

Virtual screening workflow of Glide/Schrödinger (Glide, Schrödinger, LLC, New York, NY, USA, 2018, [28,29]) was used for screening the clustered leaders. The three different conformers of GlmU were chosen and the grids were generated as discussed above. The above clustered 11,880 leader structures along with their conformers (12,23,648) were docked rigidly in all three conformers separately in three stages; HTVS followed by Glide SP, where 20% of the results and good scoring compounds were retained in both, and at Glide XP, 20% of the compounds and the best scoring compounds only were obtained.

### 3.11. E-pharmacophore Generation

The E-pharmacophore hypothesis generator module of Schrödinger [37,38] was used to generate structure-based pharmacophores. All three structures of GlmU (uridine bound to apo conformation, GlcNAc-1-P conformation and UDP-GlcNAc bound GlmU) were considered for this study. The interactions which showed consistency throughout the simulation in each of the structures were used for generating the hypothesis. Excluded volume was used during pharmacophore hypothesis generation. At the end, the pharmacophore hypothesis generated for each of the structures was merged into a single pharmacophore hypothesis while not considering redundant pharmacophore features. The merged pharmacophore hypothesis, ADAADD, was used for screening ~4.8 lacs compounds with the excluded volume option. To validate the hypothesis, the known Mtb GlmU inhibitors were randomly seeded with other screened compounds.

### 3.12. Molecular Dynamics Simulation

The simulation study was completed for Mtb GlmU structures: (a) apo conformation (PDB ID: 3D98) [6], (b) uridine modeled to apo Mtb GlmU, (c) substrate GlcNAc-1-P bound (PDB ID: 4HCQ) [8], (d) uridine modeled to GlcNAc-1-P bound Mtb GlmU conformation (e) and product UDP-GlcNAc bound conformation (PDB ID: 4G3Q).

MD simulation was carried out using the GROMACS 2019 package [39] for 20 ns. The protein force fields were generated using AMBER ff99SB [40], and GAFF (Generalized AmberForce Field) from Antechamber was used to generate force fields, topologies and parameters for ligands [41,42]. The structures were immersed in a dodecahedron box of extended TIP3P water molecules. They were neutralized by adding Na+ ions and Cl− ions. Energy minimization was performed using the steepest descent method for 50,000 steps with a 0.01 step size to relieve the short-range bad contacts. The electrostatics were examined using the particle mesh Ewald (PME) method [43,44] with short-range electrostatic cut-off and Van der Waals cut-off of 10 Å. The MD simulations production was run at 300 K temperature and 1 bar pressure, using 0.002 ps time step. The Parrinello–Rahman coupling method was used to control pressure, and V-rescale thermostat coupling was used to maintain temperature. Hydrogen bonds were constrained using the LINCS algorithm [45]. Simulations were conducted with periodic boundary conditions. The trajectories were analyzed at every 10ns to check if interactions are maintained. Furthermore, based on RMSD and RMSF, the overall complex stability was interpreted.

MD simulations for selected hits were carried out in a similar manner as above for 20 ns.

## 4. Conclusions

In this study, we aimed at targeting the Mtb GlmU Uridyltransferase site to identify potent novel inhibitors along with addressing selectivity against human homolog AGX1/AGX2. We applied two complementary computational strategies to identify inhibitors. Existing potent bacterial GlmU Uridyltransferase inhibitors were optimized based on the pocket comparisons, thereby leading to the proposal of two compounds, Compound A and Compound E. Virtual screening of the Zinc database at the Mtb GlmU Uridyltransferase substrate binding site resulted in five compounds: ZINC000015107678, ZINC000002684513, ZINC000012422035, ZINC000000485734, and ZINC000003279388. The seven compounds (two from ligand optimization and five from virtual screening) were triaged from hundreds of top ranked hits based on the key interactions and their stability during MD simulations. Moreover, for selectivity against the structurally similar human homolog, AGX1 domain, their substrate binding pockets and conformational changes upon substrate binding were analyzed and selectivity determining factors were determined. The proposed compounds are sterically hindered by the pocket residues of human homolog AGX1, thus alluding to their being selective. Overall, these Mtb GlmU Uridyltransferase domain inhibitors are predicted to be potent and selective and can serve as a starting point in the development of antibacterial against Mtb.

## Supplementary Materials

The following supporting information can be downloaded at https://www.mdpi.com/article/10.3390/molecules27092805/s1, Figure S1: Compounds from ligand optimization process, Figure S2: Binding poses of compound B, compound C, compound D, compound F, compound G and compound H, Table S1: Clustered known inhibitors, 28 from Tran et al., 2013 [2] and 7 from Soni et al., 2015 [13]; Compound ID is given as: first four digits publication year and last two digits compound number reported in the article. Highlighted in bold compounds were used as query structures for 2D similarity search, Figure S3: MD simulations of (a) Uridine docked in Apo form (3D98), (b) Uridine docked in Glc-NAc-1-P bound form (4HCQ) and (c) UD1 bound co-crystal structure (4G3Q), Figure S4a: Putative binding poses of vs. hits ZINC000015107678, ZINC000000485734, and ZINC000003279388, (b) MD simulation of ZINC000015107678, ZINC000000485734, and ZINC000003279388 for 20 ns and Figure S5: Overlay of the three vs. ZINC000015107678, ZINC000000485734, and ZINC000003279388, in human AGX1 (PDB ID: 1JV1).
