# Supplementary material for: Identification of Mtb GlmU Uridyltransferase Domain Inhibitors by Ligand-Based and Structure-Based Drug Design Approaches
Source: Molecules. 2022 Apr 28;27(9):2805. doi: 10.3390/molecules27092805 (PMC9105790; doi:10.3390/molecules27092805)

Supplementary information

Figure S1: Compounds from ligand optimization process

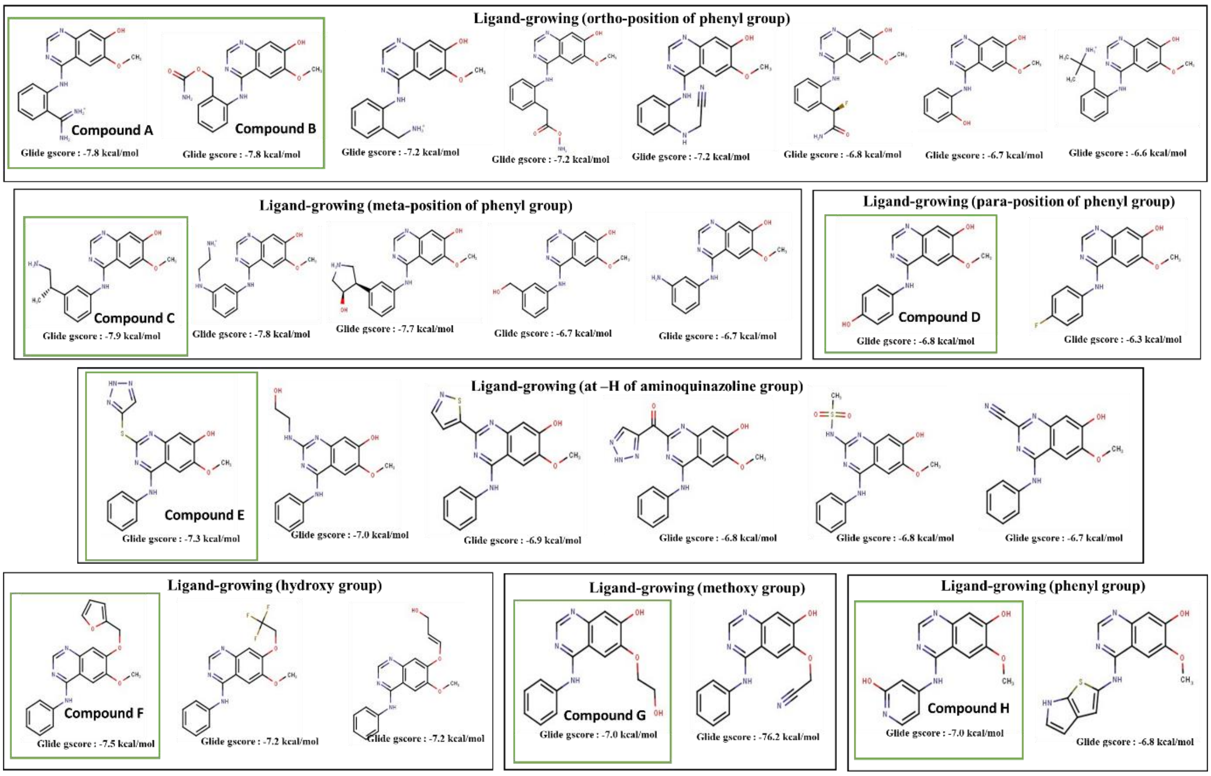

Figure S2: Binding poses of compound B, compound C, compound D, compound F, compound G and compound H

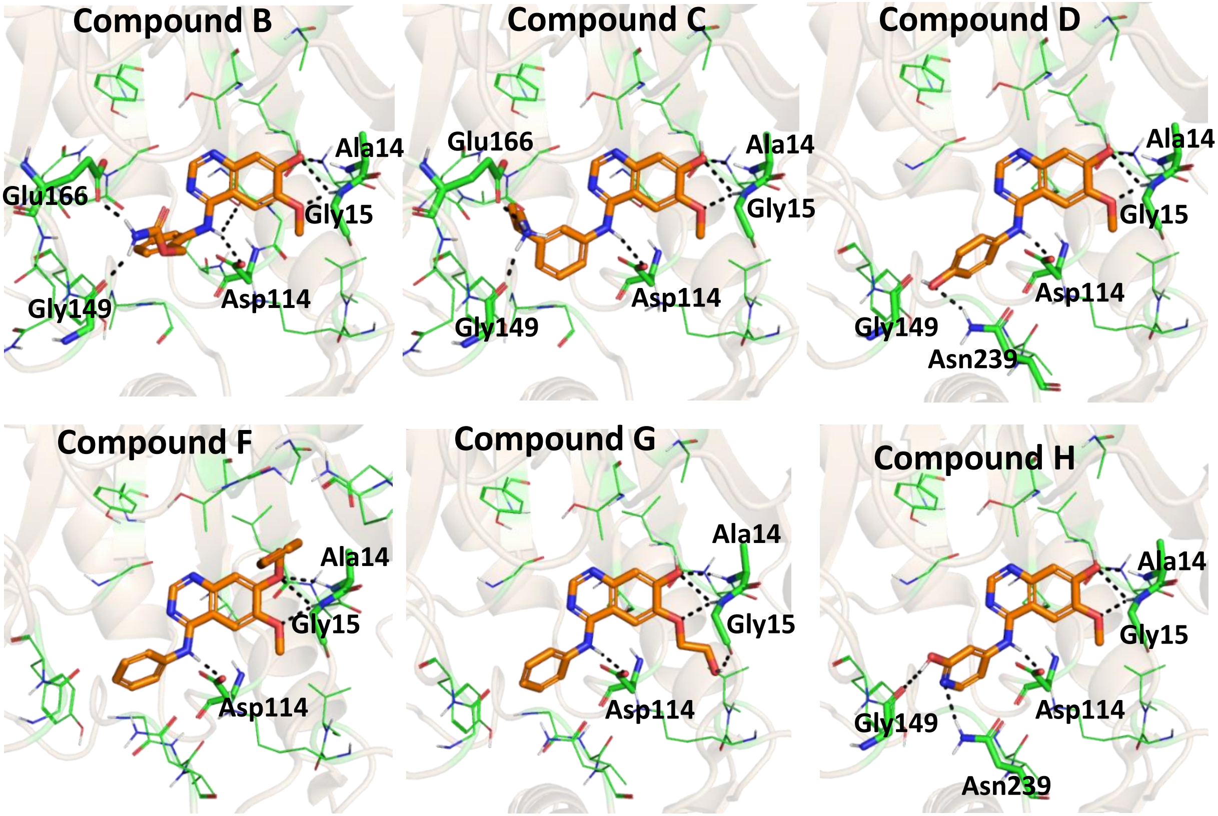

Table S1: Clustered known inhibitors, 28 from Tran et al., 2013 [2] and 7 from Soni et al., 2015 [13]; Compound ID is given as: first four digits publication year and last two digits compound number reported in the article. Highlighted in bold compounds were used as query structures for 2D similarity search

S.No.	Compound ID	Structure	% Inhibition at 50μM	Cluster
**1**	**201305**	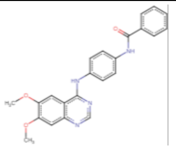	**44%**	**1**
2	201306	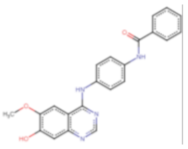	36%	1
3	201312	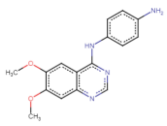	14%	1
4	201313	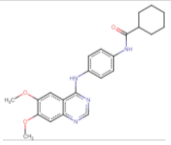	22%	1
5	201314	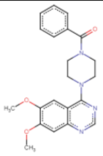	14%	1
6	201319	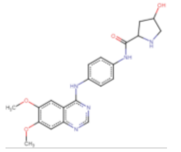	8%	1
7	201322	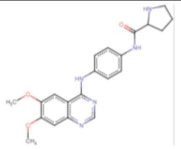	8%	1
8	201323	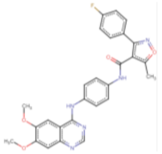	19%	1
9	201324	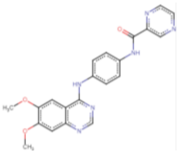	19%	1
10	201325	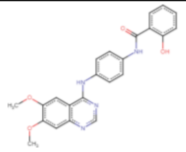	14%	1
11	201326	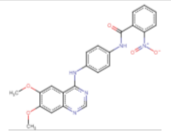	13%	1
12	201327	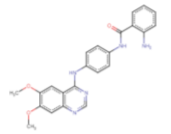	16%	1
13	201328	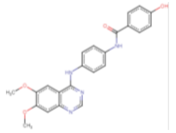	18%	1
14	201329	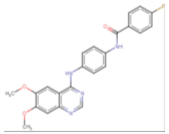	21%	1
15	201330	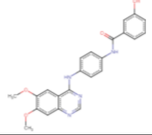	35%	1
16	201331	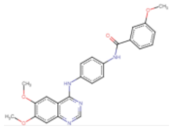	38%	1
17	201333	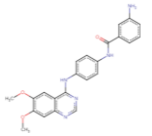	17%	1
18	201335	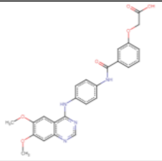	34%	1
19	201336	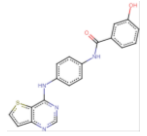	38%	1
20	201337	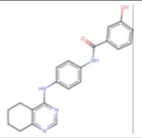	30%	1
21	201338	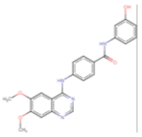	7%	1

S.No.	Compound ID	Structure	% Inhibition at 20µM	Cluster
22	201503	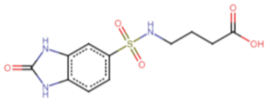	~21%	2
**23**	**201504**	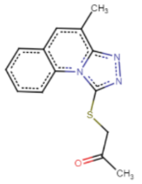	**~50%**	**3**
24	201508	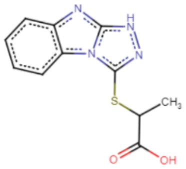	~20%	3
**25**	**201506**	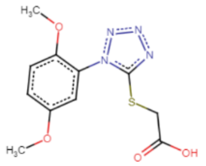	**~51%**	**4**
**26**	**201509**	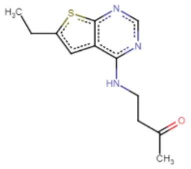	**~41%**	**5**
27	201505	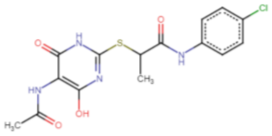	~41%	6
**28**	**201507**	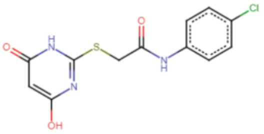	**~41%**	**6**

Figure S3: MD simulations of a) Uridine docked in Apo form (3D98), b) Uridine docked in Glc-NAc-1-P bound form (4HCQ) and c) UD1 bound co-crystal structure **(**4G3Q**)**

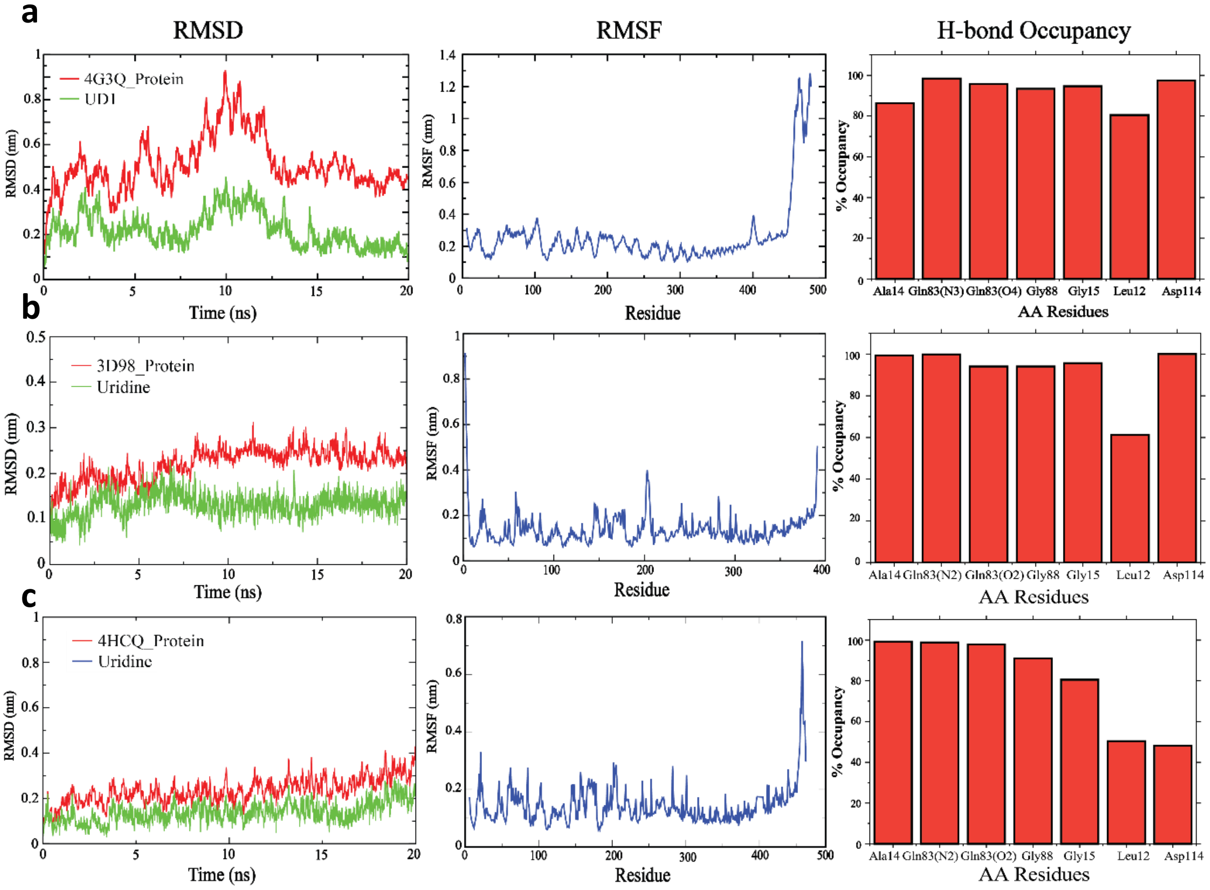

Figure S4a: Putative binding poses of VS hits ZINC000015107678, ZINC000000485734, and ZINC000003279388, b) MD simulation of ZINC000015107678, ZINC000000485734, and ZINC000003279388 for 20ns

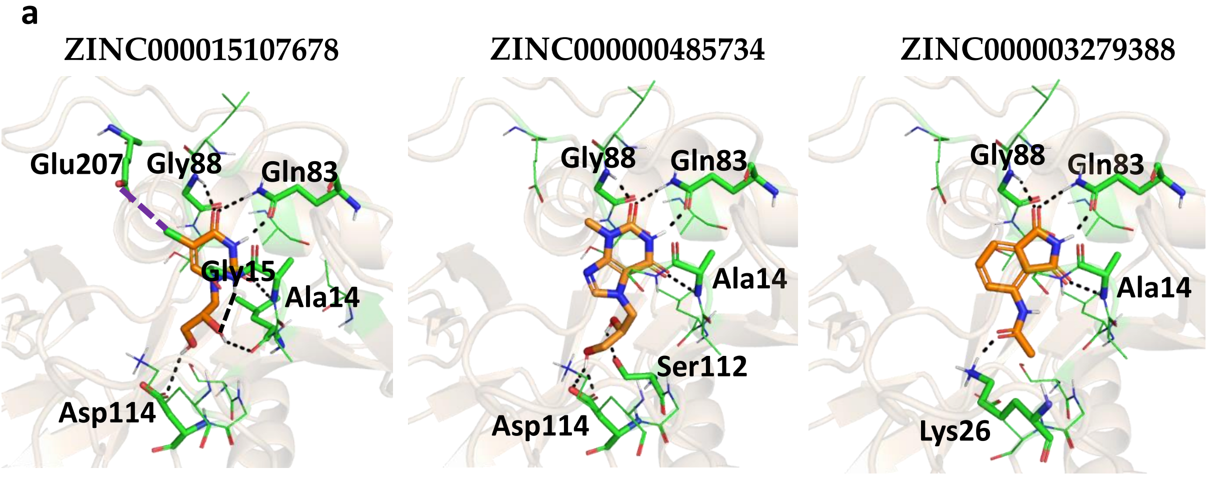

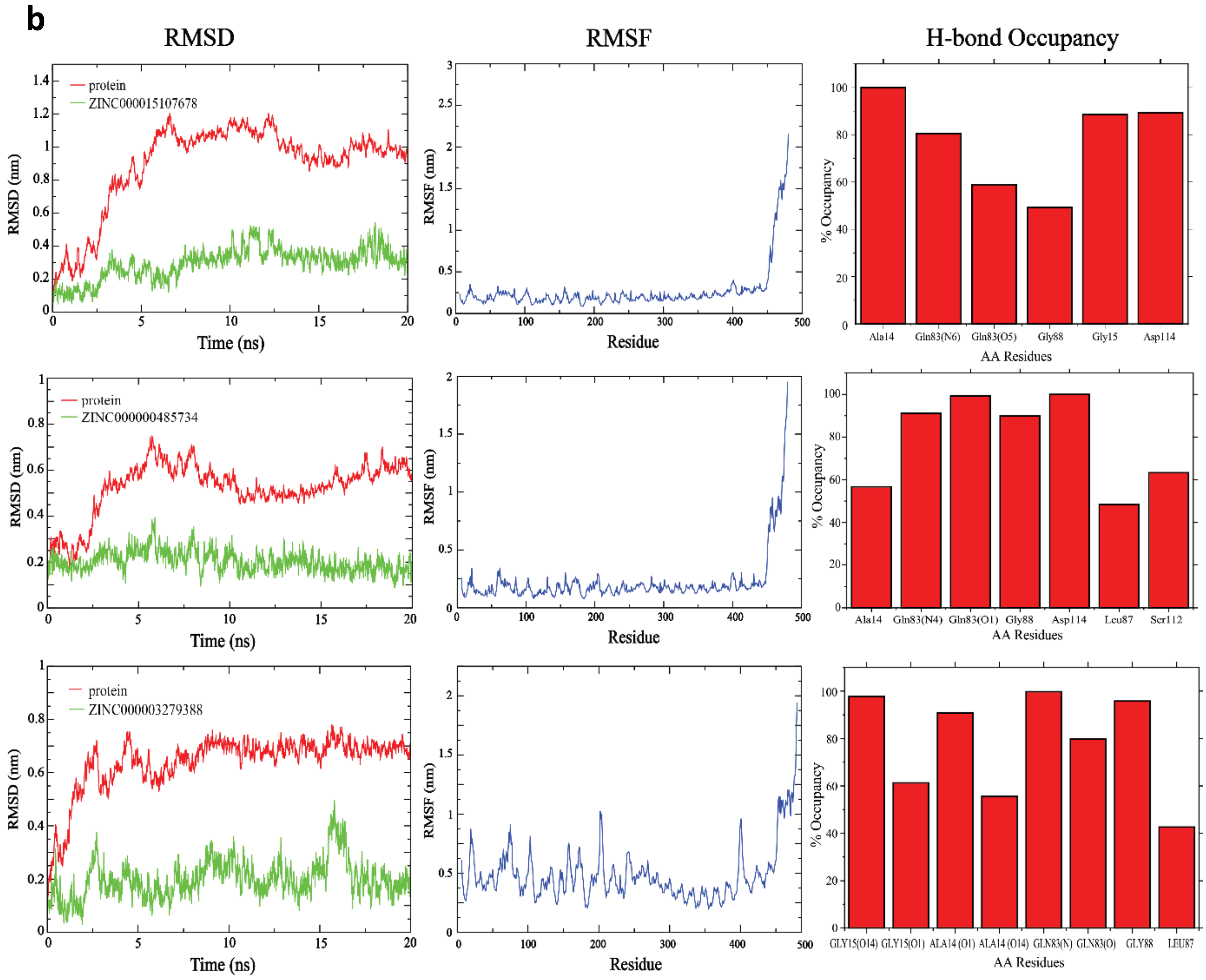

Figure S5: Overlay of the three VS ZINC000015107678, ZINC000000485734, and ZINC000003279388, in human AGX1 (PDB ID: 1JV1). Ugly clashes are shown in red color.

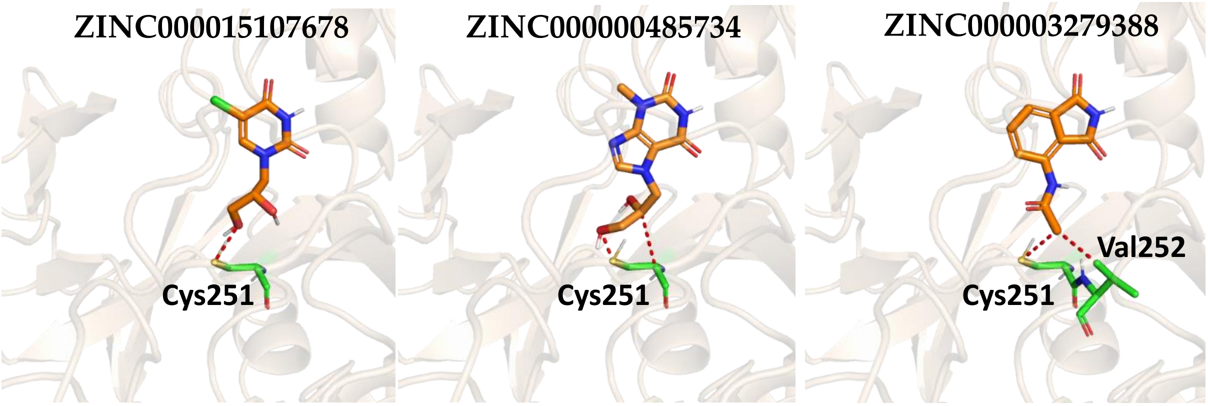

Supplement: Supplementary file 1 [file molecules-27-02805-s001.zip › molecules-1651790-supplementary.pdf]
